# Treatment of Dysmenorrhœa by Mental Suggestion or Hypnotism

**Published:** 1888-12

**Authors:** Arthur C. Hugenschmidt

**Affiliations:** (Univ. of Pa.), Paris, France


### TREATMENT OF DYSMENORRHŒA BY MENTAL SUGGESTION OR HYPNOTISM.

BY ARTHUR C. HUGENSCHMIDT, M. D.,
(Univ. of Pa.), Paris, France.

The following cases pertaining to the subject of gynæcologyst have occurred in my practice during the past year, and have been treated successfully by this method.

CASE 1.—Young woman, 27 years old, menstruated for the first time at the age of 11 years; since then her menstrual periods have been very regular, but each one of them is accompanied by a great deal of pain. Five or six days before the appearance of the flow, she has the usual unpleasant feeling described by most patients; but two days before, the pains become terrible, unbearable, the patient being compelled to lie down. The pains are situated in both ovarian regions, are very severe, and shoot down from each side into the corresponding thigh; they are accompanied by severe vomiting and headache. These symptoms last until the appearance of the flow, when the patient looses a great deal of blood, and then the pains disappear. From the train of symptoms alone some organic malformation of the uterus or displacement of the same could be expected; still I made no examination as different treatments had been tried before without success. I proposed to try the influence of mental suggestion.

This being assented to, both by the patient and her parents, I began the extremely short treatment in December last. On my first attempt to hypnotize her, I obtained no re ult by making her look at a diamond ring; on the second day, however, using the same method I obtained a better result, a light sleep, which is all that is necessary for the purpose. While the young woman was in this condition, I contradicted every one of the symptoms, which had been described to me, saying, "that she would have no more pains, no more vomiting, no more headache before, during or after her menstrual periods, that she was now entirely cured; she was then restored to consciousness.

At her next menstral period, which came on a few days later, she felt perfectly well, had no pain, n › headache, and no vomiting. The cure has pe sisted up to the present day; she has had no more return of her trouble and remains perfectly regular. In this case, these severe symptoms had lasted continuously for sixteen years; and five minutes time has been sufficient to effect a cure.

CASE II.—In May last, I was called to a young woman, 25 years old, who complained of a very severe pain in the right ovarian region; the parts were painful to pressure. She had been in that condition for two days, and could walk about the room only with great difficulty, as any movement of her right limb would increase the pain; the symptoms presented indicated a probable congestion of the right ovary.

Hypnotism was proposed as treatment and accepted; in this case, sleep was obtained by asking the patient to fix her attention upon my index finger; it took me about four minutes to place her in the proper condition, which is, as stated before, a slight hypnotic sleep. When in this state, I told her that the pain in her right side had disappeared from this moment; that she would be able to move her limb and walk about the room without any aid. On awakening, the patient stood up, began to walk, moved her right limb freely, and said she did not feel the least pain. Since then, these pains, which before would come on occasionally, have not returned.

CASE III.—A young woman, 28 years old, menstruated for the first time when 12 years old; the menstrual period is very irregular in time, but not painful. When 19 years old, without any known cause, her menses suddenly disappeared This amenorrhœa lasted one full year; then her menstruation appeared again, irregular as before, but is now accompanied at every menstrual period by severe pain and vomiting.

The symptoms were as follows: Three to five days before the flow, a general sense of lassitude, swelling of the breasts, and he ordinary signs of approaching menses; the flow then makes its appearance, and only three to four hours afterwards do these extreme pains come on. They are, as in the first case, a double ovarian pain, extremely severe, extending down both thighs; the severity of those pains produces a general coldness of the surface of the body. In addition there is severe vomiting, but no headaches; these extreme pa ns last from six to eight hours, during which time the patient remains in bed, the legs flexed on the abdomen, especially the left one. Poultices produce a certain amount of relief. I hypnotized this patient in less than two minutes, she benig a perfect subject. It is needless to say that a third person must always be present during this treatment—one of the family, especially if a young woman is the patient. As in the preceding cases, I announced the disappearance of the ovarian pains and vomiting at the next menstrual period, as well as the complete cure of the patient. Three days later, the patient menstruated, but experienced no pain whatever, and no vomiting; she felt bright and happy during the whole menstrual period. It is only two weeks since her last illness, but I have no reason to suppose that this patient will be cured in a less degre than the two preceding ones. I must add that these three patients were all well educated and very intelligent; hence the suspicion of having had to deal with persons of a low intellectual power is out of the question.

The method of treatment by mental suggestion is a very easy one, and does not, as is erroneously supposed, require a supernatural power on the part of the physician; any practitioner who will study the subject, can do as well as any man living who uses this process.—*Med. and Surg. Rep.*

